# Telephone referral education, and evidence of retention and transfer after six-months

**DOI:** 10.1186/1472-6920-12-38

**Published:** 2012-06-07

**Authors:** Stuart D Marshall, Julia C Harrison, Brendan Flanagan

**Affiliations:** 1 Southern Health Simulation Centre, Monash Medical Centre Moorabbin Campus, Centre Road, East Bentleigh, Melbourne, Australia; 2 Monash University, Academic Board of Perioperative Medicine, Commercial Road, Prahran, Melbourne, Australia; 3 University of Queensland, Cognitive Engineering Research Group, St Lucia, Brisbane, Australia; 4 Monash University, Central Clinical School, Commercial Road, Prahran, Melbourne, Australia

## Abstract

**Background:**

Effective communication between clinicians is essential for safe, efficient healthcare. We undertook a study to determine the longer-term effectiveness of an education session employing a structured method to teach referral-making skills to medical students.

**Methods:**

All final year medical students received a forty-five minute education intervention consisting: discussion of effective telephone referrals; video viewing and critique; explanation, demonstration and practice using ISBAR; provision of a memory aid for use in their clinical work. Audio recordings were taken during a subsequent standardised simulation scenario and blindly assessed using a validated scoring system. Recordings were taken immediately before (control), several hours after (intervention), and at approximately six months after the education. Retention of the acronym and self-reports of transfer to the clinical environment were measured with a questionnaire at eight months.

**Results:**

Referral clarity at six months was significantly improved from pre-intervention, and referral content showed a trend towards improvement. Both measures were lower than the immediate post-education test. The ISBAR acronym was remembered by 59.4% (n = 95/160) and used by the vast majority of the respondents who had made a clinical telephone referral (n = 135/143; 94.4%).

**Conclusions:**

A brief education session improved telephone communication in a simulated environment above baseline for over six months, achieved functional retention of the acronym over a seven to eight month period and resulted in self reports of transfer of the learning into practice.

## Background

Communication is an essential aspect of all areas of healthcare delivery. Communication skills training within undergraduate medical curricula tend to concentrate on communication with patients, with little attention paid to communication with other members of the healthcare team. The ability of health professionals to share ideas and concerns with other members of the multi-professional team is very important. Analyses of ‘sentinel events’ or critical incidents resulting in harm or death suggest that approximately 60% of these episodes are due at least partly to inadequate communication between team members [1]. Furthermore, poor communication results in many inefficiencies and frustrations that are difficult to quantify within the complexities of healthcare delivery but are recognised as commonplace in all areas of the system.

One initiative to improve inter-professional communication has been the introduction of structured communication ‘tools’ into clinical areas [2,3]. The most common tool used is based on the US Navy ‘SBAR’ technique. SBAR is an acronym that outlines four components of a communication framework: ‘Situation’, ‘Background’, ‘Assessment’ and ‘Recommendation’ [4]. The requirements of the clinical environment clearly differ from the original setting on submarines; hence the applicability of SBAR to the healthcare setting needs to be considered before widespread adoption of its use occurs. We were keen to explore the use of SBAR for telephone communication, as at our institution there have been several cases in which the wrong patient and even the wrong hospital were attended by clinicians because of poor telephone communication. We felt that it was important to introduce education on referral-making skills for final year medical students who would soon be junior doctors, as telephone referrals are a daily task for this group.

We speculated that referral-making skills become most efficient only after a clinician was also on the receiving end of referrals, however, this usually doesn’t happen for several years. We wanted to develop an education session that would accelerate the development of referral-making skills for junior doctors.

**Figure 1:**
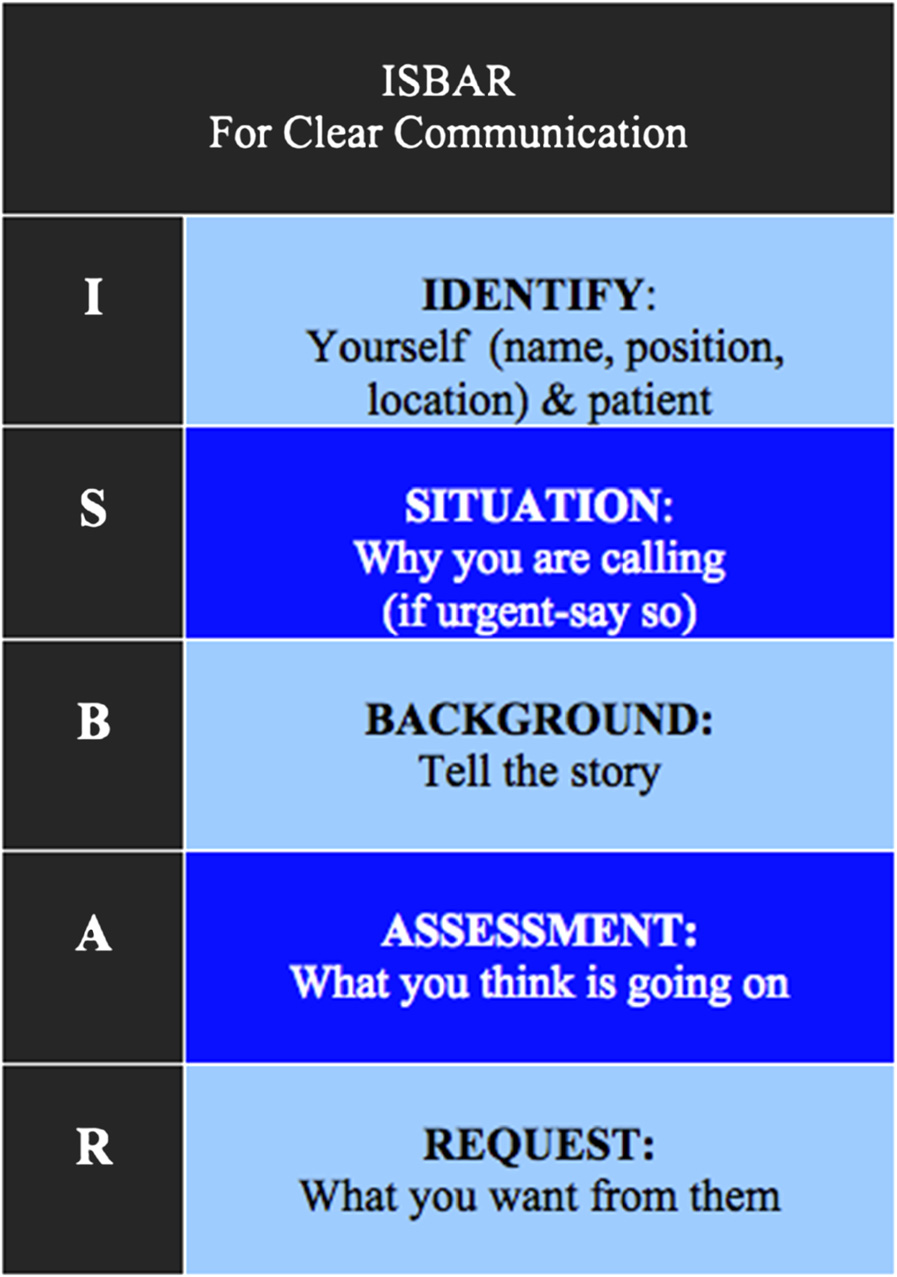
The ISBAR tool as displayed on the lanyard card.

We quickly realised that SBAR in its original form may not have prevented the miscommunication in the situations where the wrong hospital or patient was attended. As a result we separated out ‘I’ for ‘Identify’ from the ‘Situation’ element of the original tool to form an adapted version, ‘ISBAR’, shown in full as Figure 1. In addition, in the context of telephone referral, we changed the ‘R’ from ‘Recommendation’ to ‘Request’ as we were teaching students who would usually be *requesting* advice from more senior colleagues.

The use of a structured technique has two theoretical advantages: 1) junior clinicians have a structure to use in terms of the order and content of their communication that may prevent them omitting important information, and 2) clinicians who are receiving the information can anticipate receipt of the information in a standard order.

A previous study showed that both clarity and content of information are improved during telephone referral in a simulation setting after the teaching of the ISBAR tool [5]. The test scenario required multiple tasks to be performed by the team of five students, one of which was the telephone referral. Whilst this approach allowed tight control of the conditions and a close replication of the real working environment and stressors, a limitation of the study was the short duration between the education session and the testing scenario of between 2 and 4 hours.

Our aim in conducting this follow-up study was to determine if the improvement in communication was sustained, the ISBAR tool remembered, and if the students reported using the tool in their clinical placements. The interval between the education session and the follow-up testing in this study was approximately six months.

## Methods

**Figure 2:**
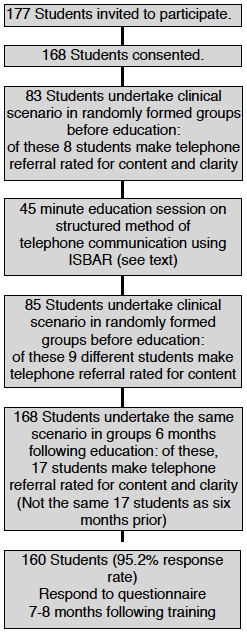
Study design.

Human Research Ethics Committee approval was obtained and all 177 students undertaking a subject on patient safety [6] were invited to participate in the study. This study was undertaken six months following an education session previously described [5] to establish how the students’ telephone communication skills had been retained and if they had used the structured approach that had been taught (Figure 2).

The 45-minute small-group education session consisted of:

A discussion of the importance of effective inter-professional communication, including telephone communication.

Video critique of two trigger video examples of ineffective telephone communication. One video demonstrating the effects of inadequate preparation and the second demonstrating a referral with too much content and lack of focus.

Introduction and explanation of a structured method of telephone communication; the ISBAR tool.

Demonstration video of ISBAR in use.

Practice using the ISBAR tool in pairs, using a paper-based scenario.

During the practice with the paper-based scenario, students had to extract information from a paper-based case, take turns making a mock telephone referral and then give each other feedback. The paper-based case involved an emergency situation of a patient requiring resuscitation and urgent gastroscopy, but much of the available information in the case was irrelevant to the immediate situation. Consequently, the students needed to analyse all the information, extract and synthesise the most immediately relevant pieces of information, and then make the referral in a structured fashion as would be required in a real situation of this kind.

At the end of the education session the students were given a lanyard card with the ISBAR tool and a brief explanation of each element on it. They were encouraged to use the card as a memory aid to reinforce the use of the ISBAR technique in their clinical placements. They were also told that they might have to make a referral using ISBAR in an Objective Structured Clinical Examination (OSCE) towards the end of the year.

The original education session was conducted on day one of a five day course on patient safety [6,7], and a previous study showed that students exposed to this teaching session before testing had higher clarity and content scores of their clinical communication [5]. Between the initial study and the follow-up, the students completed their final year clinical placements, electives, and attended the simulation centre for the remaining four days of the patient safety program at intervals throughout the year. There was no formal reinforcement of the ISBAR education session during the subsequent simulation teaching days or in the students’ clinical placements. The only reminders of the ISBAR tool were the lanyard cards and a poster positioned on the wall next to the telephone in the simulation room.

The groups of 5 or 6 students undertook the same simulation scenario as six months previously, but not in the same groups. This scenario involved the management of a patient severely injured following a high-speed car accident. In addition to gathering information, performing the primary survey and stabilising the patient, the students were expected to call for help from a senior clinician via the telephone. Although the scenario was a group activity the communication on the telephone was an individual action within that activity. The students self selected which of them made the telephone referral and were not the same students that made the calls six months earlier. One of the investigators (SM) acted as the senior clinician and gave minimal prompting to standardise the conversation as much as possible.

Audio recordings of the telephone calls were unobtrusively taken, given unique identifying codes and reviewed by another of the investigators at the end of the study period. The communication was scored for content from a list of 20 points and clarity on a 5-point rating scale with descriptors. The content score included items that were judged to be crucial for the conversation such as identifying who was calling, from where and what the vital signs were. The clarity score was a subjective scale judged against behavioural markers such as reading back important information, asking questions. Both of these scoring systems had been validated in the prior study, and a sample of the previous recordings was also reviewed to confirm continued reliability. All students had ISBAR training in the original study, however the 'control group' initially undertook the simulation scenarios prior to the training (Figure 2).

In order to determine how well the ISBAR tool was remembered, the participants also completed a questionnaire at seven to eight months following the education session. The questionnaire also covered whether the students had used the ISBAR tool to prepare or make telephone referrals during their clinical placements and what problems they encountered if using it.

Statistical analysis was performed using SPSS 15.0 (Lead Technologies), using a one-way ANOVA test for the content data and the clarity data. Questionnaire data were represented as frequency data and analysed using a chi-squared analyses for associations between demographic groups and being able to remember the acronym, and reports of using the tool. A significance level of p < 0.01 was chosen for analysis of this categorical questionnaire data due to the risk of a type I, Bonferroni error with multiple comparisons.

## Results

### Performance in simulation scenarios

A total of 19 telephone referrals were recorded at a mean period of 28 weeks after the initial education (range 19 to 37 weeks) and compared with the recordings from the previous study with a control group (prior to training) and immediate post intervention group (up to four hours post training). Two of these recordings were excluded from the analysis as the study protocol was not followed. In both of these cases the protocol breach was that an individual other than the intended investigator fielded the referral telephone call.

Reliability was adequate with a mean Cohen’s Kappa for the item scores of 0.77 (SEM 0.046) and 0.70 (SEM 0.16) for the clarity scores.

**Figure 3:**
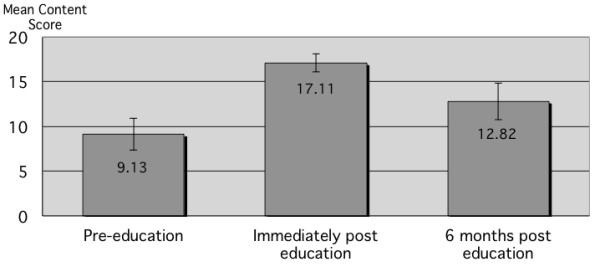
**Communication content scores of each group from a 20-item checklist.** Difference from pre-education was 7.98 for 2–4 hours post education (p < 0.001) and 3.69 at 6 months (p = 0.018). The two post education groups were also significantly different (4.29, p = 0.005). Clarity scores are given in Table 1.

Content scores for the recorded communication were significantly improved from pre-intervention, but were also significantly lower than the immediate post-education scores with means being 9.13 (SEM 0.92), 17.11 (SEM 0.51) and 12.82 (1.06) for the control, immediate and six month groups respectively (Figure 3).

Clarity scores at six months were significantly higher than the pre-intervention scores, with a trend towards lower scores, compared to immediately following the education (Table 1).

**Table 1:** Communication clarity scores

**Group**	**Mean score (SEM)**	**Kolmogorov-Smirnov test**	**Compared to control**	**Compared to immediately post-education**
**Control** (n = 8)	2.75 (0.16)	4.55 (p < 0.001)	N/A	-
**Immediately post- education** (n = 9)	4.11 (0.20)	3.51 (p = 0.002)	1.36 (0.40, p = 0.002)	N/A
**Six months after education** (n = 17, but not the same students as before)	3.47 (0.24)	2.30 (p = 0.017)	0.72 (0.35, p = 0.048)	−0.64 (0.34, p = 0.066)

Between group comparisons on the 5-point scale, are shown for before, immediately (2–4 hours) following and six months after the education session. Data were normally distributed within groups.

### Questionnaire data

A total of 160 responses were obtained to the questionnaire (95.2% response rate) 32 to 38 weeks (mean 35 weeks) following the education session. Most of the respondents were able to accurately recall the elements of the ISBAR acronym with 95 (59.4%) correct answers. Common variations are shown in Table 2.

The majority of the respondents had been given the opportunity to make a telephone referral whilst on a clinical placement (143, 89.4%), of these 135 (94.4%) had used the ISBAR tool to prepare the referral and 132 (92.3%) used the tool to make the telephone referral. Eighteen of the respondents (11.3%) experienced problems using the ISBAR tool (Table 2). No significant associations were found between the demographics of the student group, if they had or used their lanyard card and the correct recall of the acronym or use of the technique (Table 3).

**Table 2:** Questionnaire responses to the use of the ISBAR tool at seven to eight months following education

**Question**	**Response**	**Frequency of responses (%)**
Gender	M	55 (34.4)
	F	105 (65.6)
Is English your first language?	Yes	122 (76.3)
	No	38 (23.8)
Do you still have your ISBAR lanyard card?	Yes	137 (85.6)
	No	23 (14.4)
Do you routinely wear your ISBAR lanyard card?	Yes	107 (66.9)
	No	53 (33.1)
What does ISBAR stand for?	**(All 5 correct responses)**	**95 (59.4)**
	**Identify (correct)**	**122 (76.3)**
	Introduce (incorrect)	36 (22.5)
	Information (incorrect)	2 (1.3)
	**Situation (correct)**	**145 (90.6)**
	Scenario (incorrect)	4 (2.5)
	Specify (incorrect)	3 (1.9)
	**Background (correct)**	**154 (96.3)**
	**Assessment (correct)**	**154 (96.3)**
	**Request (correct)**	**128 (80.0)**
	Review (incorrect)	10 (6.3)
	Referral (incorrect)	6 (3.8)
	Reason (incorrect)	5 (3.1)
	Response (incorrect)	5 (3.1)
I have had the opportunity to make a telephone referral	Yes	143 (89.4)
	I have used ISBAR to prepare a referral	**135 (94.4)**
	I have used ISBAR to make a referral	**132 (92.3)**
I have experienced problems using ISBAR	Yes	18 (11.3)
	Interruptions	6 (3.8)
	Remembering the acronym	3 (1.9)
	Hierarchy (refuse to take referral from student)	2 (1.3)
	Need to reorder thoughts	2 (1.3)
	No further details given	5 (3.1)

**Table 3:** Associations between student demographics and lanyard card use and retention and transfer to the workplace

	**Correctly remembers ISBAR acronym (n = 160)**	**Pearson**
		**Chi-square test**
Is English your first language?	No 21 (55.3%)	0.349 (p = 0.555)
	Yes 74 (60.7%)	
Gender	M 39 (70.1%)	4.622 (p = 0.032)
	F 56 (53.3%)	
Do you still have your ISBAR lanyard card?	No 12 (52.2%)	0.577 (p = 0.447)
	Yes 83 (60.6%)	
Do you routinely wear your ISBAR lanyard card?	No 32 (60.4%)	0.033 (p = 0.856)
	Yes 63 (58.9%)	
	**Has used ISBAR format to prepare a telephone referral**	**Pearson**
		**Chi-square test**
Do you still have your ISBAR lanyard card?	No 16 (70.0%)	4.469 (p = 0.350)
	Yes 119 (86.9%)	
Do you routinely wear your ISBAR lanyard card?	No 43 (81.1%)	0.632 (p = 0.427)
	Yes 92 (86.0%)	
	**Has Used ISBAR format to make a telephone referral**	**Pearson**
		**Chi-square test**
Do you still have your ISBAR lanyard card?	No 16 (70.0%)	3.113 (p = 0.78)
	Yes 116 (84.7%)	
Do you routinely wear your ISBAR lanyard card?	No 41 (77.4%)	1.451 (p = 0.228)
	Yes 91 (85.0%)	

## Discussion

This follow-up study shows a continued effect of a 45-minute education session in improving the clarity of clinical telephone communication in a simulated clinical case. There was an expected decay in performance in both content and clarity scores compared to the groups who had undertaken the teaching just 2 to 4 hours prior to the testing. In regard to the content scores, there was a trend towards an improvement in the six month group compared to the control group, with the immediate post-education group giving significantly more information than the other two groups. This may be due to the inadequate power of the study to distinguish the smaller effect size at the six month interval compared to the immediate effect. Furthermore, the limited sample size of this study does not allow us to determine what part of the tool helps clinicians in delivering more information as it is also underpowered to determine this.

The clarity scores on the 5-point scale were significantly higher at six months than the control group, however there was an expected decay in performance compared to the students immediately after the education. The improvement in scores are most likely due to a prolonged effect of the education and the effects of its repeated practice in the clinical environment. It is unlikely to have been a result of education in the clinical environment, as few of the senior clinicians were aware of the ISBAR technique at this time. We can only speculate as to the extent to which learning about ISBAR is reinforced in the clinical environment by the students being prepared to try it.

In this study the population were fifth-year medical students; a junior group of clinicians with limited experience. The improvement of communication content and clarity in this cohort might be greater than for experienced clinicians who are experienced at making referrals, and may have less incentive to try a new format [8]. It should also be noted that the testing condition was a simulation scenario, not a real world observation. This has the advantage of controlling the ‘clinical situation’ quite tightly such that the same scenario can be presented to multiple groups, as well as allowing recording of the interaction for later blinded review without compromising patient care or confidentiality. Nevertheless, the immersive simulation environment is not the same as the real clinical environment as the cues are not the same and the stakes are different.

As in the previous study, the groups of students were divided randomly and no attempt was made to allocate the student in each group who was to perform the telephone referral. In allocating a student we would have highlighted the test condition, whereas by maintaining a naturalistic approach the telephone conversation became just one of many jobs that were required to be completed by the team. Whilst the students who were more confident in terms of telephone communication may have self-selected, this approach was followed in all of the testing scenarios equally.

The questionnaire responses at seven to eight months following the teaching session indicate that the communication tool is valued and useful in the clinical setting. The acronym was remembered by nearly 60% of respondents, a similar proportion to that shown elsewhere after initial training [9,10]. This retention rate is an encouraging sign six months after only 45 minutes of education, and seems to suggest the cognitive cueing structures associated with the mnemonic are strong [11]. These cueing structures provide a framework for remembering and organising information that is not initially recognised as related by the learner. It is noteworthy that the variations were counted as errors for purposes of this study; however, in practice they would not necessarily have affected the utility of the ISBAR tool. Cognitive aids have also been suggested to improve performance of seemingly unrelated tasks in health, particularly in stressful situations [12]. Our questionnaire did not find a relationship between retention or reported use and wearing of the ISBAR lanyard card. Further research is necessary to explore the significance of this aid and other aids to retention that could be provided in the clinical environment.

It is important to note that at the time of the study the ISBAR method was not in routine use in any of the clinical environments the students were exposed to, therefore there was little reinforcement of the learning in the clinical environment. In that respect the result is even more impressive. Moreover, it is unlikely that the majority of senior clinicians that the respondents spoke to during the telephone referrals were aware of the ISBAR tool. This may have been one contributor to the main difficulty the students had when using the tool, namely interruption by the clinician to whom they were talking. This difficulty is to be expected, and is indeed one of the drawbacks of using a structured method; that it conceptualises the communication process as a monologue rather than a dialogue.

One of the next steps in terms of this research is to develop a similar style of intervention for practicing clinicians, assessing its implementation and then measurement of outcomes in the actual clinical environment. Our organisation is in the process of attempting such an intervention.

This study was not designed to determine how much of the retention and transfer success is a result of the nature of the education session or simply because the ISBAR tool is easy to use and very helpful for junior clinicians when faced with the challenge of making a referral. We believe both the teaching and the fact that ISBAR is a good tool, are important contributors to these noteworthy results.

Aspects that we think made the education session useful are:

• As well as teaching how to make an effective referral we explained and explored why it is such an important skill; through the sharing of stories of their own experience and observations, students learn referral making is a frequent and often challenging task. This increases motivation for learning.

• Discussion of common problems with referrals, such as too much irrelevant detail, lack of preparation, lack of a clear message, not stating the “obvious” and lack of a clear question. Trigger videos and discussion were an engaging way to explore these issues.

• Active learning through the opportunity to practice, including being on both ends of the referral making process.

• Education at the right time – just as they are starting to be asked to make referrals and before they have developed bad habits.

• Practice reflection on both their own and other’s performances through practice with peers and video viewing. Hopefully this reflection on performance would continue in the clinical environment.

• Time spent learning about the referral making process may encourage students to spend more time observing and thinking about this activity in the work place.

• Being told that they may be tested on ISBAR in their final exams.

## Conclusions

A brief education session on referral skills using the communication tool ISBAR improved telephone communication skills in a simulated environment above baseline for over six months but with some decay, achieved functional retention of the acronym over a seven to eight month period and resulted in self reports of transfer of the learning into practice. The education method has been described here and we hope will provide other educators with a successful and time efficient approach to teaching referral making skills.

## Competing interests

None of the authors have any financial or non-financial competing interests to declare.

## Authors’ contributions

SDM designed the study and questionnaire, collected and analysed the data and drafted the manuscript. JCH and BF designed and delivered the education, collected the questionnaire data and helped draft the manuscript.

## Pre-publication history

The pre-publication history for this paper can be accessed here:

http://www.biomedcentral.com/1472-6920/12/38/prepub
